# Unusually Fast *bis*-Histidyl Coordination in a Plant Hemoglobin

**DOI:** 10.3390/ijms22052740

**Published:** 2021-03-08

**Authors:** Stefania Abbruzzetti, Alex J. Barker, Irene Villar, Carmen Pérez-Rontomé, Stefano Bruno, Giulio Cerullo, Cristiano Viappiani, Manuel Becana

**Affiliations:** 1 Dipartimento di Scienze Matematiche, Fisiche e Informatiche, Università di Parma, Parco Area delle Scienze 7/A, 43124 Parma, Italy; stefania.abbruzzetti@unipr.it; 2 Center for Nano Science and Technology@PoliMi, Istituto Italiano di Tecnologia, via G. Pascoli 70/3, 20133 Milano, Italy; alex.barker@iit.it; 3 Departamento de Nutrición Vegetal, Estación Experimental de Aula Dei, Consejo Superior de Investigaciones Científicas (CSIC), Apartado 13034, 50080 Zaragoza, Spain; ivillar@eead.csic.es (I.V.); rontome@eead.csic.es (C.P.-R.); 4 Dipartimento di Scienze degli Alimenti e del Farmaco, Università di Parma, Parco Area delle Scienze 27/A, 43124 Parma, Italy; stefano.bruno@unipr.it; 5 IFN-CNR, Dipartimento di Fisica, Politecnico di Milano, Piazza Leonardo da Vinci 32, 20133 Milano, Italy; giulio.cerullo@fisi.polimi.it

**Keywords:** *Medicago truncatula*, ultrafast spectroscopy, plant hemoglobins, CO rebinding kinetics, iron/heme hexacoordination

## Abstract

The recently identified nonsymbiotic hemoglobin gene *MtGlb1-2* of the legume *Medicago truncatula* possesses unique properties as it generates four alternative splice forms encoding proteins with one or two heme domains. Here we investigate the ligand binding kinetics of MtGlb1-2.1 and MtGlb1-2.4, bearing two hemes and one heme, respectively. Unexpectedly, the overall time-course of ligand rebinding was unusually fast. Thus, we complemented nanosecond laser flash photolysis kinetics with data collected with a hybrid femtosecond–nanosecond pump–probe setup. Most photodissociated ligands are rebound geminately within a few nanoseconds, which leads to rates of the bimolecular rebinding to pentacoordinate species in the 10^8^ M^−1^s^−1^ range. Binding of the distal histidine to the heme competes with CO rebinding with extremely high rates (*k_h_* ~ 10^5^ s^−1^). Histidine dissociation from the heme occurs with comparable rates, thus resulting in moderate equilibrium binding constants (*K_H_* ~ 1). The rate constants for ligation and deligation of distal histidine to the heme are the highest reported for any plant or vertebrate globin. The combination of microscopic rates results in unusually high overall ligand binding rate constants, a fact that contributes to explaining at the mechanistic level the extremely high reactivity of these proteins toward the physiological ligands oxygen, nitric oxide and nitrite.

## 1. Introduction

Plant hemoglobins were first discovered in the root nodules of legumes. They were termed leghemoglobins and shown to be able to oxygenate reversibly. Subsequently, hemoglobins were also found in the nodules of actinorhizal plants and *Parasponia* (for a review of early references, see [1]). The only known function of leghemoglobins and other symbiotic hemoglobins of nodules is the transport and delivery of O_2_ to the nitrogen-fixing bacteria [1]. However, we know now that hemoglobins are widespread in nonsymbiotic tissues of all plants (for reviews, see [2,3,4,5,6,7]). These nonsymbiotic hemoglobins (now called phytoglobins (Glbs)) can be categorized into three classes according to phylogenetic analyses and biochemical properties [6,7,8,9,10]. Class 1 Glbs have an extremely high affinity for O_2_ and weak hexacoordination of the iron heme with a distal His (His_d_) residue, whereas class 2 Glbs have strong hexacoordination which results in O_2_ affinities that are ~100-fold lower than those of class 1 Glbs [10]. In both Glb classes, the presence of hydrophobic cavities and tunnels modulates ligand exchange between the active site on the heme distal site and the solvent, providing preferential pathways for the supply of reactants and egression of products [11,12,13,14]. Class 3 Glbs show high sequence similarity with bacterial truncated hemoglobins and relatively low O_2_ affinity [8,10,15]. The class 3 Glb of *Arabidopsis thaliana* (AtGlb3) is hexacoordinate when deoxygenated, but this state is transitory and reverts slowly (20 min) to the pentacoordinate form [15]. However, only a few Glbs of classes 2 and 3 have been characterized at the biophysical level.

The genes of the three Glb classes may respond differently to environmental conditions. Class 1 Glb genes are upregulated by hypoxia, osmotic and cold stress, nutrient deprivation, rhizobial and fungal infection and nitric oxide (NO) [6,16,17,18,19,20]. As shown for the class 1 Glbs of monocots and *A. thaliana* (AtGlb1), a function of class 1 Glbs is to modulate NO concentration, which allows the plant to adapt and survive under limiting O_2_ conditions [3]. This is thought to be due in part to the NO dioxygenase activity of the proteins, whereby the oxyferrous globin reacts with NO to yield nitrate and ferric globin [21,22]. Much less is known about the functions of class 2 and 3 Glbs. The class 2 Glb of *A. thaliana* (AtGlb2) is responsive to low temperatures and cytokinin treatment [23,24]. This protein also reacts with NO in vivo [22,25] and is involved in plant morphogenesis [25] and in the regulation of somatic embryogenesis [19]. Class 3 Glbs are unresponsive to several types of stress that affect the other two Glb classes, although they also respond to the supply of certain hormones in the roots [18]. A function for these Glbs in NO metabolism has been proposed in the symbiosis of plants with rhizobia and arbuscular mycorrhizal fungi [26]. In fact, a subclass of class 3 Glbs (Glb3-1) are highly expressed in nodules [18,27].

The recently identified *MtGlb1-2* gene of the model legume *Medicago truncatula* is unique because it generates four alternative splice forms, designated *MtGlb1-2.1* to *MtGlb1-2.4*, encoding proteins with one or two heme domains [28]. Thus, MtGlb1-2.1 and MtGlb1-2.2 have two heme domains, whereas MtGlb1-2.4 has one heme domain and it is unclear whether MtGlb1-2.3 has one or two heme domains. The *MtGlb1-2* gene is transcriptionally activated by hypoxia and NO and is preferentially expressed in the meristematic region and vascular bundles of roots and root nodules [28]. In this work, we have used CO as a model ligand to investigate the binding properties of MtGlb1-2.1 and MtGlb1-2.4, as representative proteins with two hemes and one heme, respectively. These Glbs are extremely reactive toward the physiological ligands O_2_, NO and nitrite and show very high O_2_ affinities, NO dioxygenase activity (in the presence of O_2_) and nitrite reductase activity (in the absence of O_2_). A detailed kinetic characterization of the ligand rebinding processes required extension of temporal resolution to the short picosecond range, with the use of a hybrid femtosecond–nanosecond pump–probe setup. The results show unprecedented properties of these Glbs, with very large and fast geminate rebinding and very high bimolecular CO rebinding rates and His_d_ binding and dissociation rates. Our studies with His_d_ mutants confirmed the identity of the kinetic processes attributed to the His_d_ binding and dissociation.

## 2. Results and Discussion

### 2.1. Unusually Fast CO Rebinding Kinetics to WT1 and WT4 after Nanosecond Laser Photolysis

**Figure 1:**
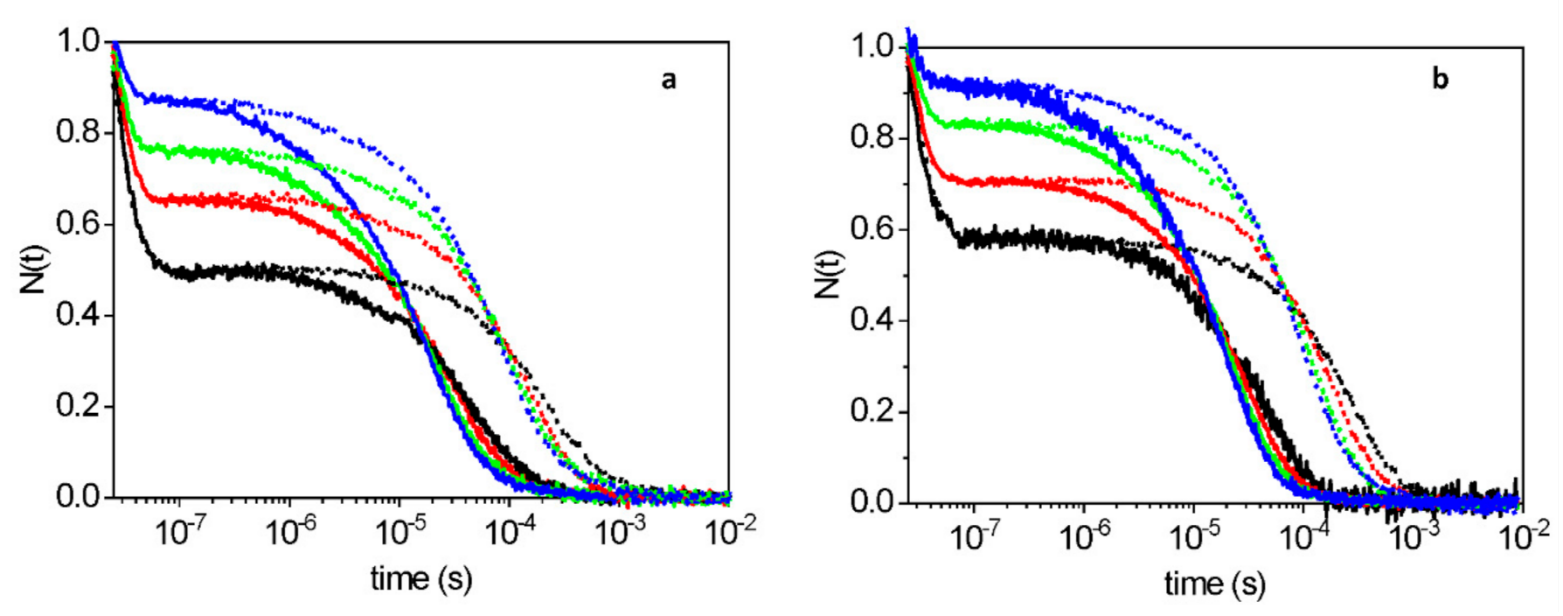
CO rebinding kinetics after nanosecond laser flash photolysis (LFP) of (**a**) WT1 and (**b**) WT4 solutions equilibrated with 1 atm CO (solid lines) and 0.2 atm CO (dotted lines) at 10 °C (black), 20 °C (red), 30 °C (green) and 40 °C (blue). Data are reported as the progress curve representing the fraction of deoxy molecules, N(t), as a function of time after photolysis. The absorbance change was monitored at 436 nm, following laser photolysis at 532 nm.

The CO rebinding kinetics to wild-type MtGlb1-2.1 (WT1) and MtGlb1-2.4 (WT4) after nanosecond laser flash photolysis (LFP) are qualitatively very similar and are shown in Figure 1 for temperatures between 10 °C and 40 °C and for solutions equilibrated with 1 or 0.2 atm CO. As previously reported for other hexacoordinate hemoglobins, the overall progress curve is composed of two well-separated processes for both proteins. A fast, CO concentration-independent (unimolecular) rebinding is evident on the short nanosecond time scale, followed by a CO concentration-dependent (bimolecular) rebinding phase on the microsecond time scale. The effect of different CO concentrations at each temperature is easily appreciated by comparing the solid (1 atm CO) and the dotted (0.2 atm CO) curves.

The faster phase is commonly interpreted as unimolecular ligand rebinding from within the protein matrix and is called geminate rebinding. A minor amplitude decay is detectable in the long nanoseconds to short microseconds, whose CO concentration dependence is very weak. In contrast, the slower process occurring in the microsecond time scale shows a CO concentration dependence, indicating a bimolecular nature. Decreasing CO concentration results in slower and broader microsecond rebinding kinetics, best described by double exponential relaxations especially at higher temperatures (Figure S1, Table S1). Considering that the two proteins in their ferrous state show *bis*-histidyl hexacoordination [28], the heterogenous kinetics in the microsecond time scale is thus expected to reflect competitive distal histidine (His_d_) binding to, and dissociation from, the unliganded Fe^2+^ after photolysis, as previously reported for other plant and vertebrate hemoglobins [29,30,31]. The slowest portion of the kinetics is usually attributed to His_d_ dissociation, and the amplitude of this process increases with temperature and upon decreasing CO concentration.

The qualitative effect of temperature on CO rebinding kinetics is easily appreciated by comparing rebinding curves at the same CO concentration in Figure 1. The first striking feature is that the amplitude of geminate rebinding increases dramatically upon decreasing the temperature, at the expense of the amplitude of the microsecond rebinding. Since the binding step to the heme is normally characterized by a very low enthalpic barrier, this finding is indicative of a large activation energy to exit from the protein matrix and reach the solvent. The microsecond process shows an increase in apparent rate at higher temperatures, directly revealing a thermal activation of the associated rate constant. Unlike other Glbs, the time required for His_d_ dissociation and ligand rebinding completion is extraordinarily short (~1 ms). For comparison, other hexacoordinate Glbs from plants are characterized by His_d_ dissociation rates spanning from 10 s^−1^ (class 2 Glbs) to 10^2^ s^−1^ (class 1 Glbs) [10]. The His_d_ dissociation rates from vertebrate hexacoordinate hemoglobins are much lower (~0.1–1 s^−1^) [5].

Importantly, the smaller than expected absorbance change at the end of the laser pulse and the steep drop of the signals in the short (nanoseconds) timescales (Figure 1) suggest that a relevant portion of the rebinding kinetics may fall within the 7 ns laser pulse width. Thus, in order to be able to perform a quantitative kinetic analysis, experiments with improved time resolution are necessary to complete the time course of the rebinding processes, accessing microscopic steps that occur in the subnanosecond time range.

### 2.2. Experiments with Subnanosecond Resolution Reveal a Large Geminate Rebinding

We thus applied a hybrid femtosecond–nanosecond pump–probe setup to collect the full time course of CO rebinding and gain access to the subnanosecond time frame. Besides allowing subnanosecond resolution, the setup allows collecting the kinetics up to 1 ms, seamlessly merging the ultrafast timescale with the time range normally covered in nanosecond LFP experiments. This feature of the apparatus represents a potentially major improvement over a more traditional approach, leaving a gap between the longest time accessible in femtosecond pump–probe (~1–2 ns) and the shortest time measured with nanosecond LFP (~20–30 ns) [32,33,34]. The proposed setup represents an alternative experimental approach that is simpler than previously reported instruments based on two independent and electronically synchronized femtosecond lasers [35,36].

**Figure 2:**
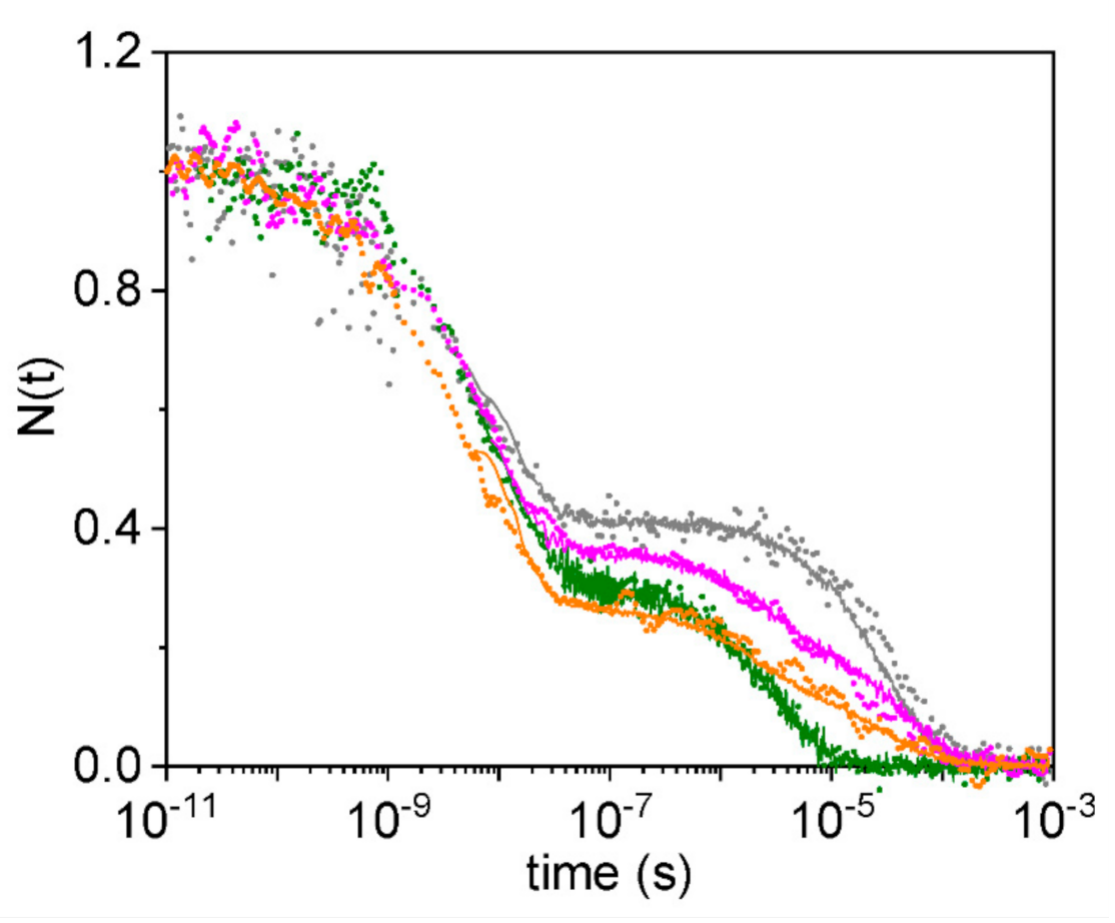
CO rebinding kinetics for WT1 (gray dots) solution equilibrated with 1 atm CO and at room temperature collected with the hybrid femtosecond–nanosecond pump–probe setup. The corresponding kinetics measured after nanosecond LFP is reported as a gray solid line. For comparison, the rebinding kinetics to single and double His_d_ mutants are reported (orange, 238; magenta, 74; olive, 74/238; see Section 2.4 for definitions).

Figure 2 compares the CO rebinding kinetics for WT1 at room temperature collected with the hybrid femtosecond–nanosecond pump–probe setup (gray dots) and with the nanosecond LFP setup (gray solid line). From simple visual inspection, it is evident that there is perfect agreement between the two curves in the time range where they overlap. Excellent agreement was also observed for MtGlb1-2.1 single and double mutants at the His_d_ (see Section 2.4). Similar results were obtained for WT4 and its His_d_ mutant.

The overall progress curve shows that a relevant fraction of CO rebinding occurs in the picoseconds, with a geminate phase accounting for 60% of the kinetics in WT1 (Figure 2). Similarly, the amplitude of geminate phase to WT4 is found to be ~70% of the overall rebinding. Thus, <50% of the photodissociated ligands in WT1 and WT4 escape geminate rebinding and migrate to the solvent, giving rise to the bimolecular phase.

Geminate rebinding to WT1 and WT4 is characterized by a biexponential kinetics (the two faster kinetic phases for each curve shown in Figure S2 and Table S2). The source of this heterogeneity may be related to either the presence of two heme domains with different binding rate constants (for WT1) or the presence of migration to nearby docking sites, from which the ligand is then rebound. Similar behavior was reported for geminate rebinding to truncated hemoglobins for which the identification of ligand egression and migration pathways from molecular modeling gave support to kinetic modeling of observed rebinding traces in some truncated globins from bacteria [32,33,37,38] and for nitrophorin 7 [39]. We favor the ligand migration interpretation for both WT1 and WT4 because the second-order rebinding is described by a single rate constant (see Supplementary Materials).

### 2.3. Description of the Rebinding Kinetics with a Microscopic Model

**Scheme 1:**
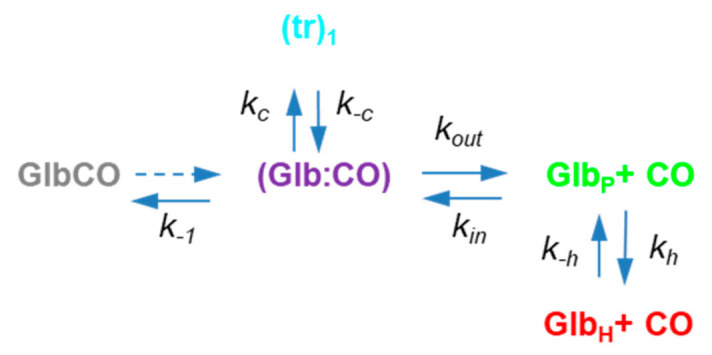
Minimal kinetic scheme proposed for the reaction of CO with Glbs. Deoxy (pentacoordinate) and *bis*-histidyl (hexacoordinate) species are indicated by Glb_P_ and Glb_H_, respectively. Immediately after photodissociation of GlbCO, the ligand is in a primary docking site (Glb:CO) within the distal pocket. From this site, the ligand can be rebound (rate *k_−1_*), migrate to a nearby temporary docking site (tr)_1_ with rate *k_c_* (reverse rate *k_−c_*), or exit to the solvent with rate *k_out_*. Ligands are rebound by Glb_P_ from the solvent with rate *k_in_*, whereas binding and dissociation of His_d_ occur with rates *k_h_* and *k_−h_*.

In the light of the above considerations, we modeled the observed kinetics using a reaction scheme (Scheme 1), where the unimolecular geminate phase comprises migration to a temporary docking site, accessible from the distal pocket, and the slower phase is the result of competitive binding to the heme of CO and the His_d_. The numerical method has been previously discussed [32,37,40].

**Figure 3:**
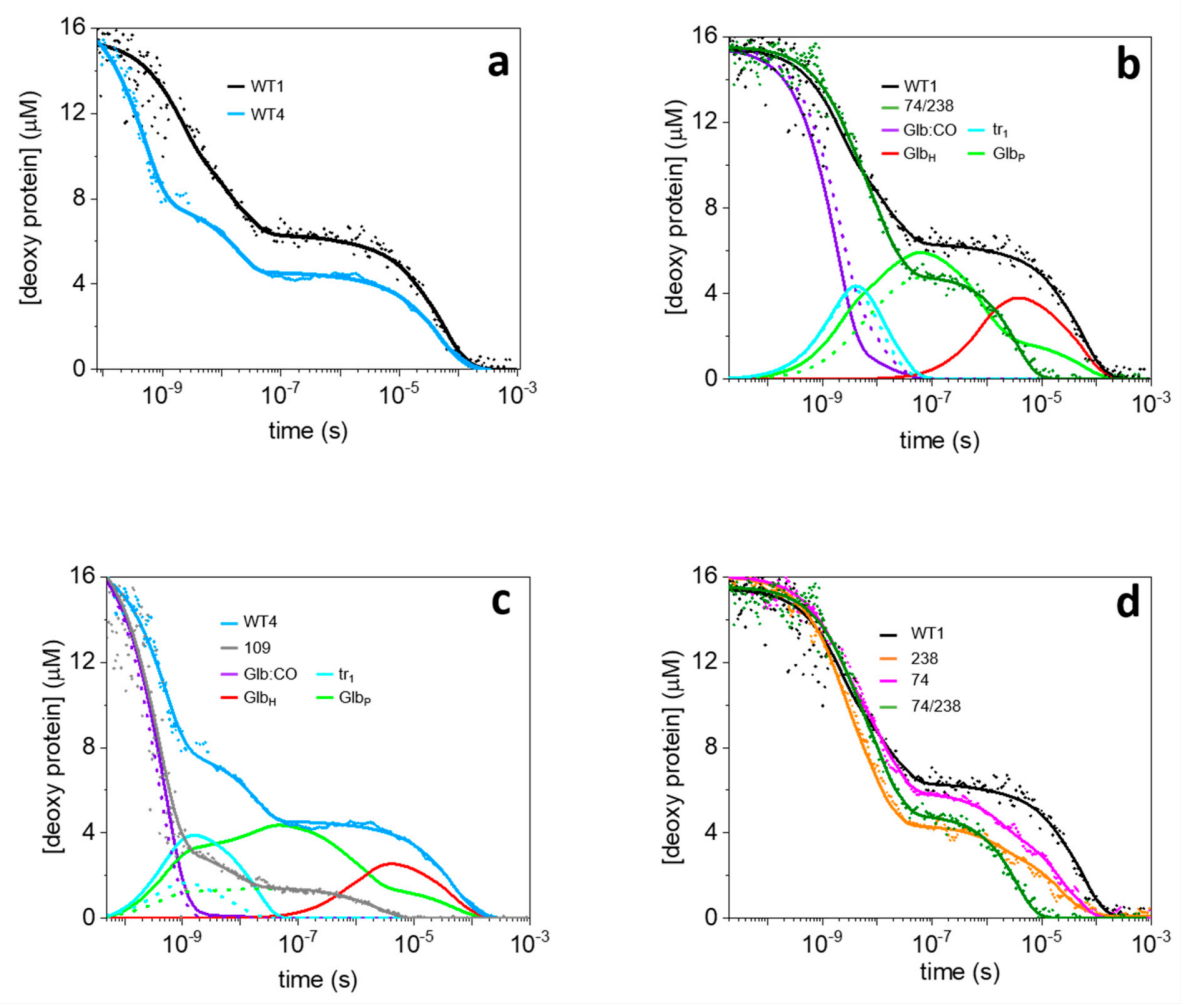
Analysis of the complete course of CO rebinding kinetics to WT1, WT4 and their mutants. The progress curve for the CO rebinding reaction after the hybrid pump–probe experiment is plotted as concentration of deoxy heme versus time. (**a**) Comparison between WT1 and WT4. The experimental data are superimposed on the fitted curves obtained using Scheme 1. (**b**,**c**) Replacement of His_d_ by Leu affects the time evolution of the CO rebinding kinetics, as demonstrated by the comparison between WT1 and its mutant 74/238 in (**b**) and between WT4 and its mutant 109 in **c**. The fitting curves are superimposed on the experimental data (circles). In (**b**,**c**), the time courses of the other relevant species are shown using the same color code as for the kinetic model reported in Scheme 1. (**d**) Comparison between the kinetics of WT1 and its 74, 238 and 74/238 mutants.

The agreement between the experimental data and the fitting curves built with the numerical model is satisfactory, as can be appreciated in Figure 3a for WT1 and WT4. For simplicity, in this panel, we plotted the modeled rebinding kinetics on top of the measured kinetics. In Figure 3b–d we also report the time course of the reaction intermediates. The values of the microscopic rates obtained in the global analysis of data at 0.2 and 1 atm CO at room temperature are reported in Table 1.

The microscopic rate constant values retrieved from the fitting of pump–probe data using the minimal kinetic scheme proposed in Scheme 1 can be used to calculate the CO binding rate constant to the pentacoordinate species (*k_on_* = *k_−1_k_in_*/(*k_out_* + *k_in_*)), the observed CO binding rate (*k_obs_* = *k_on_*[CO]*k_−h_*/(*k_−h_* + *k_h_* + *k_on_*[CO])) and the His_d_ equilibrium binding constant (*K_H_ = k_h_*/*k_−h_*).

For both WT1 and WT4, the large amplitude kinetics in the picosecond–nanosecond scale (Figure 3b,c) is described through a significant direct CO rebinding to ferrous heme from a primary docking site in the distal cavity (*k_−1_* ~10^8^ s^−1^ for WT1 and ~10^9^ s^−1^ for WT4), modulated by a transient population (*k*_c_ and *k*_−c_ ~10^8^ s^−1^ for both proteins) of a secondary docking site, accessible from the distal pocket. The large amplitude of geminate rebinding in WT1 and WT4 suggests that the connection between the distal heme pocket and the solvent is inhibited in favor of the direct rebinding or internal migration. The innermost binding rate (*k_−1_*) shows values for WT1 and WT4 (Table 1) that are much higher than the ones reported for other hexacoordinate heme proteins, such as human neuroglobin (1.5 × 10^7^ s^−1^ [41,42]) and the Glbs from *A. thaliana* (0.5 × 10^7^ s^−1^ for AtGlb1 and 2.2 × 10^7^ s^−1^ for AtGlb2 [12]) or from rice (Osat1-1; 7.6 × 10^7^ s^−1^ [31]), and similar only to the ones typical of truncated HbO (3 × 10^8^ s^−1^ for *Thermobifida fusca* [32], 8.37 × 10^8^ s^−1^ for *Pseudoalteromonas haloplanktis* TAC125 [37], ~8 × 10^8^ s^−1^ for truncated HbO from *Mycobacterium tuberculosis* [43]). Direct rebinding through the very high rate (*k_−1_*) is only partially reduced by the competitive migration to the inner docking site (*k_c_* = 2.3 × 10^8^ s^−1^ for WT1 and 6.8 × 10^8^ s^−1^ for WT4) and exit to the solvent (*k_out_* = 1.4 × 10^8^ s^−1^ for WT1 and 4.9 × 10^8^ s^−1^ for WT4). It is worth noting that the secondary docking site in WT4 provides a more stable kinetic trap to photodissociated ligands than in WT1 and results in a distinct kinetic step on the 10 ns time scale (Figure 3a, blue trace). The low fraction of photodissociated ligands reaching the solvent is mostly determined by the very high rates for direct rebinding and internal migration rather than by a low exit rate. Indeed, comparison with typical *k_out_* values for class 1 nonsymbiotic Glbs like AtGlb1 (9 × 10^7^ s^−1^ [44]) and rice (OsGlb1-1, 7.33 × 10^7^ s^−1^ [31]) shows that the exit rate for WT1 is 2-fold higher, whereas *k_out_* for WT4 is almost one order of magnitude larger. However, in those cases, the internal rates were much lower.

The structural and dynamical determinants for this behavior may be identified when structural information becomes available. Unfortunately, a topological description of cavities in the three-dimensional structure of WT1 and WT4 is not yet available, and it is not possible to identify the structural nature of the kinetic trap. However, the presence of cavities and migration pathways for ligands in hexacoordinate hemoglobins is well established in other plant Glbs, such as OsGlb1-1 [31] and AtGlb1 and AtGlb2 [12], as well as in vertebrate hemoglobins like human neuroglobin [41,45] and cytoglobin [46]. Stable or transient cavities and tunnels are usually lined by hydrophobic amino acids. Thus, they can accommodate diatomic gaseous ligands and function as kinetic traps [47]. A role of the kinetic traps in support of multisubstrate reactions, such as NO dioxygenase activity, has been suggested for myoglobin and hexacoordinate hemoglobins [48].

### 2.4. Rate Constants for His_d_ Binding and Dissociation Are Extraordinarily High

A surprising result of the numerical analysis is the very high rate constants for association (*k_h_* = 7–8 × 10^5^ s^−1^) and dissociation (*k_−h_* = 3–4 × 10^5^ s^−1^) of the His_d_ (Table 1), which are about three orders of magnitude larger than the reported values for any class 1 Glb (average *k_h_* = 130 s^−1^ and *k_−h_* = 75 s^−1^) or class 2 Glb (average values *k_h_* = 1500 s^−1^ and *k_−h_* = 25 s^−1^) [10], with unusually low energetic barriers (vide infra, Table 2). Moreover, His_d_ dissociation rates from vertebrate hexacoordinate globins are much lower (~0.1–1 s^−1^) [5], and the highest *k_h_* value known so far is 1.4 × 10^4^ s^−1^ and comes from a mollusk hexacoordinate hemoglobin [49]. Nevertheless, the resulting equilibrium binding constant of His_d_ (*K_H_*) of ~2 (Table 1) to ferrous heme is in line with class 1 Glbs (average 1.7 ± 0.8) [5]. The microscopic rate constants enable us to calculate the binding rate constants to the pentacoordinate species as follows in Equation (1)

(1) $$k=\;\frac{k_{-1}k_{in}}{k_{in}+\;k_{out}}$$

yielding *k_on_* = 1 × 10^8^ M^−1^s^−1^ for WT1 and *k_on_* = 2.4 × 10^8^ M^−1^s^−1^ for WT4. These values are at least one order of magnitude larger than for other class 1 nonsymbiotic plant Glbs (average value: (8.4 ± 14) × 10^6^ M^−1^ s^−1^) [10]. A practical consequence of the very high dissociation rates is that the observed CO binding rate, Equation (2)

(2) $$k=\frac{k_{on}\left[CO\right]k_{-h}}{k_{on}\left[CO\right]+\;k_{-h}+\;k_{h}}$$

falls in the 10^4^ s^−1^ range (2.6 × 10^4^ s^−1^ for WT1 and 6 × 10^4^ s^−1^ for WT4) at 1 atm CO (Table 1).

In order to prove that the slowest kinetic step in the progress curve is associated with the formation of a *bis*-histidyl species, we obtained mutants in which the His_d_ is replaced by Leu in one of the two heme domains of MtGlb1-2.1 (mutants 74 and 238) or in the two domains (mutant 74/238). Likewise, we replaced the His_d_ with Leu in the single heme domain of MtGlb1-2.4 (mutant 109). Then, we examined the CO rebinding of the mutated proteins. The resulting progress curves of the 74/238 (Figure 3b) and 109 (Figure 3c) mutants show larger geminate rebinding. This indicates that the His_d_ mutation interferes with ligand migration to internal cavities and to the solvent. On longer time scales, the bimolecular phase becomes monoexponential, confirming the identification of the His_d_ dissociation with the slowest kinetic step in the WT proteins.

Comparisons of the best fit curves obtained for CO rebinding kinetics between WT1 and 74/238 (Figure 3b) or between WT4 and 109 (Figure 3c) show that the experimental data for the mutants can be described using the same kinetic scheme used for the WT proteins but without the kinetic step leading to the formation of the *bis*-histidyl species (Glb_H_, red traces). The amplitude of the geminate phase becomes larger in the mutants with respect to the WT proteins, particularly for the 109 mutant. This effect is the result of changes in *k_−1_*, *k_out_* and *k_in_.* The innermost binding rate *k_−1_* increases ~2-fold in the 109 mutant and *k_out_* decreases by a similar factor, whereas *k_in_* increases ~3.5-fold and ~6.5-fold in the 74/238 and 109 mutants, respectively.

These results suggest a possible role of His_d_ in modulating ligand exchange with the exterior and in modifying the plasticity of the protein structure. The *k_on_* rate remains practically unchanged in the 74/238 mutant but increases by a factor of 7 in the 109 mutant.

### 2.5. Dissecting the Contribution of the His_d_ in Each Domain of MtGlb1-2.1

Figure 3d shows the effect of eliminating only one His_d_ in MtGlb1-2.1. It is evident that the kinetics for single mutants are intermediate between those of WT1 and 74/238. The geminate amplitude becomes slightly larger in the 74 mutant and even more so in the 238 mutant. It is evident that the effect of the His_d_ on the heme reactivity of each domain is similar but not identical. This observation is in line with the different nitrite reductase activity recently demonstrated by some of us for the two mutants [28].

**Figure 4:**
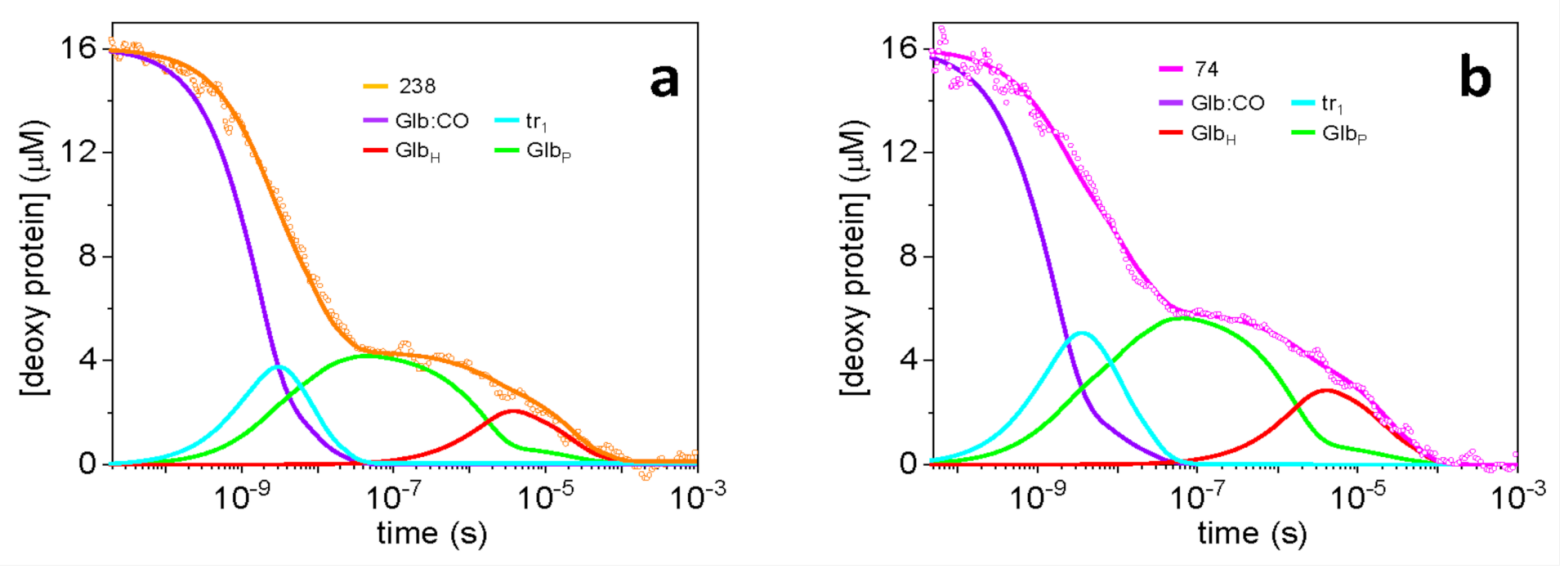
Analysis of CO rebinding kinetics to the (**a**) 238 and (**b**) 74 mutants at room temperature, 1 atm CO. The fitting curves are superimposed on the experimental data (open circles). The time course of the relevant species shown in the kinetic scheme (Scheme 1) are reported: Glb:CO (violet), (tr)_1_ (light blue), Glb_H_ (red) and Glb_P_ (green).

The kinetic analyses, according to Scheme 1, are shown in Figure 4, and the fitting parameters are listed in Table 1. With respect to WT1, there are no substantial differences in the rate constants that describe ligand migration to the kinetic trap and to the solvent, whereas CO rebinding from the outside appears to be more influenced by mutations (~2.5-fold change in rate constant). According to these parameters, *k_on_* increases from 1 × 10^8^ M^−1^ s^−1^ for WT1 to 1.5 × 10^8^ M^−1^ s^−1^ for the 74 mutant and to 2 × 10^8^ M^−1^ s^−1^ for the 238 mutant. The effect of these single mutations on *k_on_* confirms that accessibility of the distal cavity is influenced by His_d_, as already suggested for the 74/238 mutant. On the basis of these results, we propose that when the His_d_ is replaced by a Leu residue, a more accessible exchange channel between the distal pocket and the solvent is opened, through which CO molecules can migrate in and out. As expected, due to the fact that MtGlb1-2.1 bears two hemes, the amplitude of the hexacoordinate species is partly reduced, but not removed, by single His_d_ mutations (74 and 238). Moreover, the rate constants for ligation and deligation in single mutants are smaller than the corresponding WT1 values: change in *k_h_* ~2.5-fold for both mutants; change in *k_−h_* ~2.9-fold for the 74 mutant and 2.3-fold for the 238 mutant. This suggests that the single mutations affect the dynamics of the other domain and result in increased rate constants for His_d_ binding and dissociation, with little consequences on the equilibrium binding constants *K_H_* = *k_h_*/*k_−h_*.

### 2.6. Thermal Activation of Kinetic Processes

Finally, we examined the temperature dependence of CO rebinding kinetics in the range of 10–40 °C (Figure 1). It is evident that, upon increasing temperature, the amplitude of the geminate phase decreases and the bimolecular phase speeds up. The former consideration indicates that protein fluctuations significantly assist CO migration inside the protein and the exchange between inside and outside, whereas the latter evidences the typical thermal activation behavior for diffusion-mediated processes. Kinetics acquired at the same temperature at 1 and 0.1 CO atm were simultaneously analyzed in order to improve the reliability of the best fit parameters. Each global analysis permits the estimation of the microscopic rate constants of the observed processes at a defined temperature. Based on previous studies on related Glbs and other hemoglobins [32,33,37], we assume in the analysis that the innermost rate constants *k_−1_*, *k_c_* and *k_−c_* are independent of temperature. From the temperature dependence of the microscopic rate constants, the activation energy of each kinetic step can be evaluated from the resulting linear Arrhenius plots (Figure S3). The values are reported in Table 2 for WT proteins and their mutants. Because only the temperature dependence of laser LFP traces can be used, only the thermal activation of the bimolecular phase and the hexacoordination process were studied. The exit rate to the solvent (*k_out_*) has an energy barrier similar for WT1 and WT4 (>10 kcal/mol), which is significantly higher than the values reported for AtGlb1 (2.7 kcal/mol [11]) and OsGlb1-1 (4.3 kcal/mol [31]). By contrast, the entry rate from the solvent (*k_in_*) shows an activation energy in line with the values reported for AtGlb1 (14.7 kcal/mol [11]) and OsGlb1-1 (12.4 kcal/mol [31]). The energy barrier associated with *k_out_* is only half as large for the mutants. The decrease of the thermal activation energy of *k*_in_ is smaller in WT1 single mutants and is ~2-fold for the 109 and 74/238 mutants, where no His_d_ residue is present. These results are in agreement with the ones retrieved from the analysis of pump–probe experiments on the mutants that suggest a more accessible pathway between the distal pocket and the solvent when the His_d_ is substituted by a Leu residue. Interestingly, thermal activation associated with ligation (*k_h_*, 1.1 kcal/mol for WT1 and 1.4 kcal/mol for WT4) and deligation (*k_−h_*, 5.1 kcal/mol for WT1 and 2.6 kcal/mol for WT4) is heavily reduced with respect to AtGlb1 and OsGlb1-1 and other hexacoordinate globins in general. In fact, the energy barriers are 19 kcal/mol (AtGlb1) and 15.5 kcal/mol (OsGlb1-1) for *k_h_*, whereas values of 18 kcal/mol (AtGlb1) and 14 kcal/mol (OsGlb1-1) were retrieved for *k_−h_*. This low thermal activation is in line with the kinetic results that highlight very high reaction rates for His_d_ binding and dissociation (*k_h_* and *k_−h_*).

## 3. Conclusions

The two splice forms, *MtGlb1-2.1* and *MtGlb1-2.4*, of the recently identified *MtGlb1-2* gene of the model legume *M. truncatula* encode proteins with two heme domains and one heme domain, respectively. Both are extremely reactive toward the physiological ligands O_2_, NO and nitrite and show very high O_2_ affinities, NO dioxygenase activity (in the presence of O_2_) and nitrite reductase activity (in the absence of O_2_). Ligand rebinding kinetics of the model diatomic ligand CO reveal unusual properties that may be fundamental to sustain the high reactivity toward multisubstrate reactions. Both proteins show unprecedentedly high rate constants for His_d_ binding and dissociation, which results in moderate equilibrium binding constants, typical of class 1 Glbs. A very high rebinding rate to the heme Fe is responsible for an unusually large and fast geminate recombination, which mostly occurs in the subnanosecond time scale. The high binding rate to the heme results in very high bimolecular binding rates. As a result of the above properties, the observed binding rate of the diatomic ligands is extremely large and may be at the basis of the extreme reactivity of the proteins in the multisubstrate reactions they are involved in.

## 4. Materials and Methods

### 4.1. Protein Expression and Purification

The coding regions of *MtGlb1-2.1* and *MtGlb1-2.4* [28] were synthesized with codon optimization for expression in *Escherichia coli*. Gene synthesis and site-directed mutagenesis were performed by GenScript (Hong Kong). The MtGlb1-2.1 and MtGlb1-2.4 proteins and their mutant derivatives were cloned into the pET30a (+) expression vector (Novagene, Madison, WI, USA) with an N-terminal His-tag. The proteins were expressed in *E. coli* C41(DE3) cells (Lucigen, Middleton, WI, USA) and purified on nickel-affinity columns (HiTrap Chelating HP; GE Healthcare, Uppsala, Sweden) as previously described [28].

### 4.2. Nanosecond Laser Flash Photolysis of CO Rebinding Kinetics

Measurements were carried out using LFP instrumentation very similar to that described previously [12]. Briefly, the second harmonic of a nanosecond Nd:YAG laser (Surelite I-10, Continuum, San Jose, California, USA) was used to photodissociate CO molecules, and a continuous-wave probe beam from a 75 W Xe lamp was exploited to monitor transient absorbance changes at 436 nm. The time evolution of transmitted intensity was followed on a digital scope and then converted into absorbance change, ∆A (t). The temperature of the sample was precisely controlled with a Peltier element (Flash 100, Quantum Northwest, Inc. Liberty Lake, USA), allowing temperature stability better than 0.1 °C.

### 4.3. Ultrafast Spectroscopy of CO Rebinding Kinetics

Transient absorption experiments were performed in transmission geometry. An amplified Ti:sapphire laser (Integra-C, Quantronix, East Setauket, NY, USA) generated 100-fs pulses centered at 800 nm, at a repetition rate of 1 kHz. A broadband UV-Vis probe was generated by focusing the pulses into a thin CaF_2_ plate mounted on a moving stage to avoid laser-induced damage. For time delays <1 ns, the sample was excited with pump pulses centered at 530 nm (~100 fs FWHM), generated in a home-built noncollinear parametric amplifier [50] with pump–probe delay determined by an optical delay line. For delays >1 ns, pump pulses centered at 532 nm (700 ps FWHM) were obtained from a Q-switched Nd:YVO_4_ laser (Picolo, InnoLas, Krailling, Germany) which was electronically triggered and synchronized to the Ti:sapphire laser via an electronic delay [51]. After interaction with the sample, a spectrometer (Shamrock 303i Andor, Belfast, United Kingdom) dispersed the probe light onto a fast CCD array, enabling broadband shot-to-shot detection of differential absorption (∆A) spectra.

### 4.4. Data Analyses

The complete time evolution of CO rebinding kinetics was analyzed using a minimal kinetic model as previously described for other plant Glbs [11]. Numerically solving the differential equations associated with the scheme with the function ODE15s within MATLAB (MathWorks, Natick, MA, USA) and optimizing the parameters with a MATLAB version of the optimization package Minuit (CERN) allowed us to evaluate the microscopic rate constants of the observed processes.

## Figures and Tables

**Table 1:** Microscopic rate constants from the fit of pump–probe data at room temperature.

Rate Constants	Units	MtGlb1-2.1				MtGlb1-2.4	
		WT1	74	238	74/238	WT4	109
*k_−1_*	×10^8^ s^−1^	2.0 ± 0.4	1.9 ± 0.4	2.6 ± 0.5	1.6 ± 0.3	14 ± 3	23 ± 4
*k_h_*	×10^5^ s^−1^	8 ± 1	3.8 ± 0.7	4.2 ± 0.8	-	7 ± 1	-
*k_−h_*	×10^5^ s^−1^	3.8 ± 0.7	1.3 ± 0.3	1.6 ± 0.3	-	3.4 ± 0.7	-
*k_out_*	×10^8^ s^−1^	1.4 ± 0.3	1.1 ± 0.2	0.9 ± 0.2	0.7 ± 0.1	5 ± 1	2.1 ± 0.4
*k_in_*	×10^8^ M^−1^s^−1^	1.4 ± 0.3	3.7 ± 0.7	3.4 ± 0.7	4.8 ± 0.9	1.0 ± 0.2	6.5 ± 0.9
*k_c_*	×10^8^ s^−1^	2.3 ± 0.4	2.9 ± 0.6	2.5 ± 0.5	2.3 ± 0.4	6.8 ± 0.9	3.3 ± 0.7
*k_−c_*	×10^8^ s^−1^	1.2 ± 0.2	1.8 ± 0.4	2.7 ± 0.5	2.5 ± 0.5	0.8 ± 0.2	1.7 ± 0.3
*k_on_*	×10^8^ M^−1^s^−1^	1.0 ± 0.2	1.5 ± 0.3	2.0 ± 0.4	1.4 ± 0.3	2.4 ± 0.4	17 ± 3
*K_H_*		2.2 ± 0.4	2.9 ± 0.6	2.5 ± 0.5	-	2.0 ± 0.4	-
*k_obs_*	×10^4^ s^−1^	2.6 ± 0.5	2.7 ± 0.5	3.7 ± 0.7	-	6 ± 1	-

**Table 2:** Activation energies (kcal/mol) determined from Arrhenius plots in the temperature range 10–40 °C. Microscopic rate constants are retrieved from the fit of LFP data.

Rate Constants	MtGlb1-2.1				MtGlb1-2.4	
	WT1	74	238	74/238	WT4	109
*k_out_*	10.7 ± 0.4	8.2 ± 0.2	7.0 ± 0.5	6.1 ± 0.2	11 ± 1.0	4.1 ± 10.7
*k_in_*	13.6 ± 0.9	11.7 ± 0.1	11 ± 1.0	7.8 ± 0.3	14 ± 2.0	-
*k_h_*	1.1 ± 0.2	2.7 ± 1.4	4.8 ± 0.5	-	1.4 ± 0.4	-
*k_−h_*	5.1 ± 0.2	4.3 ± 0.6	3.4 ± 0.8	-	2.6 ± 0.2	-

## Supplementary Materials

The following are available online at https://www.mdpi.com/1422-0067/22/5/2740/s1. Figure S1: CO rebinding kinetics using nanosecond LFP in function of CO concentration at 40 °C. Figure S2: Analysis of the complete course of CO rebinding kinetics to WT1 and WT4. Figure S3: Arrhenius plots for the rate constants obtained from the analysis of nanosecond LFP data of WT1 and WT4. Table S1: Lifetimes from the fit of nanosecond LFP data at 40 °C at 1 atm and 0.1 atm CO for WT1 and WT4 and their mutants, using a sum of exponential decay functions. Table S2: Lifetimes from the fit of hybrid pump–probe data at room temperature at 1 CO atm for WT1 and WT4, using a sum of exponential decay functions.

## Abbreviations

Glb	Phytoglobin (or nonsymbiotic hemoglobin)
His_d_	Distal histidine
LFP	Laser flash photolysis
NO	Nitric oxide
O_2_	Molecular oxygen
CO	Carbon monoxide
WT	Wild type
