# Supplementary material for: Unusually Fast bis-Histidyl Coordination in a Plant Hemoglobin
Source: Int J Mol Sci. 2021 Mar 8;22(5):2740. doi: 10.3390/ijms22052740 (PMC7962945; doi:10.3390/ijms22052740)
Supplement: Supplementary file 1 [file ijms-22-02740-s001.pdf]

Supplementary Information

The time evolution of the transient absorption change after nanosecond LFP is well described by a four exponential decays function over the entire temperature range investigated (10-40 °C). Selected curves at 40 °C are reported in Figure S1. Parameters retrieved from the fitting at 40 °C are reported in Table S1. The first transient, in the nanosecond range, is independent of CO concentration and is thus associated with CO geminate rebinding. The lifetime (11 ns for WT1 and 12 ns for WT4 at 20 °C) reproduces well that obtained with a fitting of the complete temporal courses retrieved with the hybrid pump-probe setup (see Table S2). The small amplitude transient observed in the short microseconds shows little, if any, dependence on CO concentration, and it is possibly associated with a conformational relaxation of the protein following CO photodissociation. The next exponential step shows a clear CO concentration dependence, demonstrating its bimolecular rebinding nature. Interestingly, we observe only one step with the expected CO concentration dependence, thus we conclude that even for the two-heme WT1 a single apparent rate is observed. The value of the apparent rate should be considered as an average between the (likely similar) values for the two domains. Finally, the longer lived transient shows some CO concentration dependence. We note that at 40 °C the fractional amplitude increases from 6.8% to 9.2% for WT1 and from 2.3% to 7.8% for WT4, for solutions equilibrated with 1 and 0.2 atm CO, respectively. This finding is consistent with the association of this step to a conformational change triggered by photolysis. Based on the available experimental data, this intermediate is identified as the *bis*-histidyl hexacoordinated species. We note that, even if the hybrid pump-probe set up drastically improves the temporal resolution and widens the temporal window with respect to nanosecond LFP, the kinetics we obtained did not enhance the S/N ratio. The consequence of this fact is that small amplitude signals are difficult to retrieve from the expanded kinetics. We observe that the small amplitude signals in nanosecond LFP experiments become much smaller in amplitude in the hybrid pump-probe experiment, since >60% of rebinding occurs geminately.

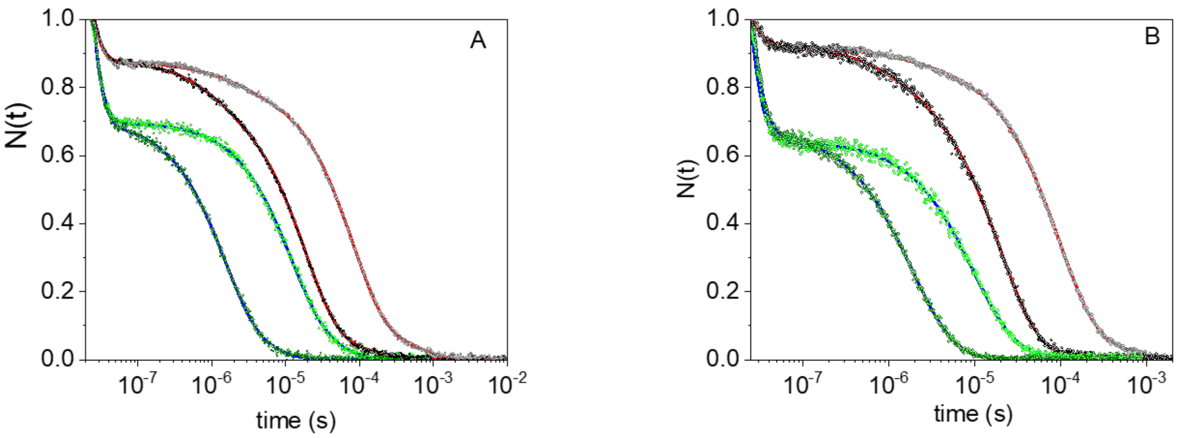

Figure S1. CO rebinding kinetics using nanosecond LFP as a function of CO concentration at 40 °C. The fraction of deoxy heme (N(t)) is plotted as a function of time. **Panel A**. Fitting of the progress curve for CO rebinding to WT1 solution (*black circles*, 1 atm CO; *gray circles*, 0.2 atm CO) and its double His_d mutant, 74/238 (*dark green circles*, 1 atm CO; *light green circles*, 0.2 atm CO). Fitting curves (*red lines* for WT1 and *blue lines* for the double mutant) are superimposed to the data. **Panel B**. Fitting of the progress curve for CO rebinding to WT4 (*black circles*, 1 atm CO; *gray circles*, 0.2 atm CO) and its His_d mutant, 109 (*dark green circles*, 1 atm CO; *light green circles*, 0.2 atm CO). Fitting curves (*red lines* for WT4 and *blue lines* for the mutant) are superimposed to the data.

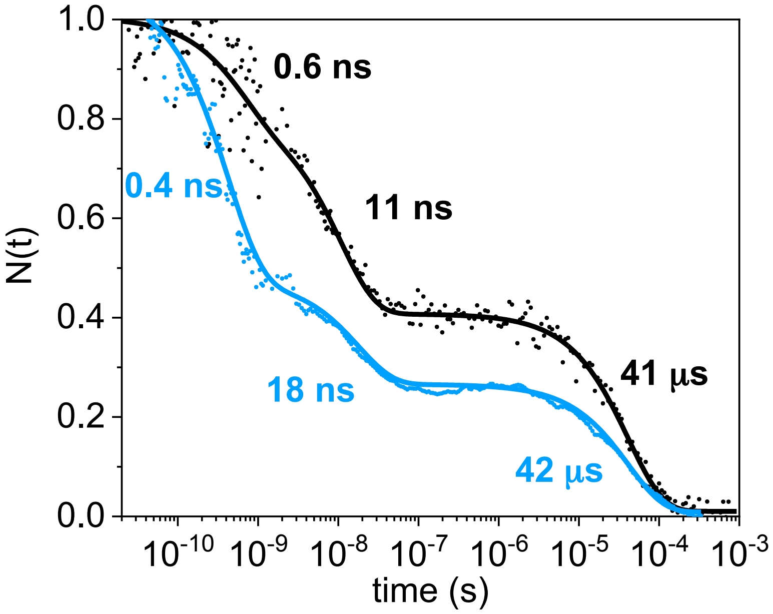

Figure S2. Analysis of the complete course of CO rebinding kinetics to WT1 (*black*) and WT4 (*blue*). The progress curve for the CO rebinding reaction after hybrid pump-probe experiment is plotted as fraction of deoxy heme as a function of time. The fitting curves (*solid lines*) obtained using a sum of three exponential decay functions are superimposed to the experimental data (*circles*). Experiments were performed at room temperature and 1 atm CO.

Table S1 Lifetimes from the fit of nanosecond LFP data at 40 °C at 1 and 0.2 atm CO for WT1 and its 74/238 mutant and for WT4 and its 109 mutant, using a sum of exponential decay functions. The fractional amplitudes for the transients are reported in parentheses.

		**Geminate phase**		**Bimolecular phase**	**Histidine deligation**
		**τ_1 (ns)**	**τ_2 (μs)**	**τ_3 (μs)**	**τ_4 (μs)**
**WT1**	1 atm	6.8±0.2 (25%)	1.03±0.03 (9.7%)	18.0±0.1 (58.5%)	118±7 (6.8%)
	0.2 atm	6.8±0.2 (25%)	1.94±0.7 (6.3%)	78.9±0.5 (59.5%)	565±22 (9.2%)
**74/238**	1 atm	7.5±0.1 (46%)	1.41±0.03 (44%)	5.3±0.5 (10%)	
	0.2 atm	7.5±0.1 (46%)	10.7±0.3 (41%)	37±3 (13%)	
**WT4**	1 atm	8.68±0.03 (15.1%)	1.2±0.1 (7.6%)	19.8±0.1 (75%)	219±41 (2.3%)
	0.2 atm	8.68±0.03 (14.8%)	1.9±0.1 (3.7%)	93±0.1 (73.7%)	884±60 (7.8%)
**109**	1 atm	7.74±0.01 (26%)	1.5±0.1 (46%)	3.2±0.3 (28%)	
	0.2 atm	7.74±0.01 (26%)	8.6±0.4 (56%)	21±3 (18%)	

Table S2 Lifetimes from the fit of hybrid pump-probe data at room temperature at 1 atm CO for WT1 and WT4, using a sum of exponential decay functions. The fractional amplitudes for the transients are reported in parentheses.

	**Geminate phase**	**Geminate phase**	**Bimolecular phase**
	**τ_1 (ns)**	**τ_2 (ns)**	**τ_3 (μs)**
**WT1**	0.6±0.1 (20%)	11±1 (40%)	41±3 (40%)
**WT4**	0.41±0.01 (54%)	18±1 (20%)	42±2 (26%)
